# Patterns of clinical response in patients with alopecia areata treated with ritlecitinib in the ALLEGRO clinical development programme

**DOI:** 10.1111/jdv.20547

**Published:** 2025-02-17

**Authors:** B. King, P. Mirmirani, K. Lo Sicco, Y. Ramot, R. Sinclair, L. Asfour, K. Ezzedine, C. Paul, M. Ohyama, R. A. Edwards, G. Bonfanti, U. Kerkmann, D. Wajsbrot, R. Ishowo‐Adejumo, S. H. Zwillich, A. Lejeune

**Affiliations:** ^1^ Dermatology Physicians of Connecticut Fairfield Connecticut USA; ^2^ The Permanente Medical Group Vallejo California USA; ^3^ The Ronald O. Perelman Department of Dermatology New York University Grossman School of Medicine New York New York USA; ^4^ Department of Dermatology Hadassah Medical Center Jerusalem Israel; ^5^ The Faculty of Medicine Hebrew University of Jerusalem Jerusalem Israel; ^6^ Sinclair Dermatology Melbourne Victoria Australia; ^7^ The Dermatology Centre Salford Royal Hospital Salford UK; ^8^ Department of Dermatology Henri Mondor University Hospital and EpiDermE Paris France; ^9^ Department of Dermatology Toulouse University and CHU Toulouse France; ^10^ The Department of Dermatology Kyorin University Faculty of Medicine Tokyo Japan; ^11^ Health Services Consulting Corporation Boxborough Massachusetts USA; ^12^ Engineering Ingegneria Informatica Milan Italy; ^13^ Pfizer Pharma GmbH Berlin Germany; ^14^ Pfizer, Inc New York New York USA; ^15^ Pfizer, Inc Collegeville Pennsylvania USA; ^16^ Pfizer, Inc Groton Connecticut USA; ^17^ Pfizer, Inc Paris France

## Abstract

**Background:**

Ritlecitinib, an oral JAK3/TEC family kinase inhibitor, demonstrated efficacy over 48 weeks in patients with alopecia areata (AA) in the ALLEGRO phase 2b/3 study.

**Objectives:**

This post hoc analysis evaluated individual Severity of Alopecia Tool (SALT) score trajectories in patients who received ritlecitinib 50 mg and rolled over from Phase 2b/3 into the ongoing, open‐label, Phase 3 ALLEGRO‐LT study to describe long‐term response patterns and associated baseline disease characteristics.

**Methods:**

Patients aged ≥12 years with ≥50% scalp hair loss received ritlecitinib 50 mg once daily in both studies. SALT score trajectories from baseline to Month 24 were used to categorise patients as early (SALT score ≤20 at Week 24 and Months 12 and 24), middle (≤20 at Months 12 and 24) or late responders (≤20 by Month 24) or as partial responders (maintained 30% improvement), relapsers (achieved but did not maintain 30% improvement) or non‐responders (did not achieve 30% improvement). The proportions of patients achieving sustained response (achieved and maintained SALT score ≤20 at all subsequent available time points through Month 24) and complete response (SALT score 0 at ≥1 time point through Month 24) were evaluated. Multivariable logistic regression assessed variables associated with response.

**Results:**

Of 191 patients treated with ritlecitinib 50 mg, 87 (45.5%) were responders (SALT score ≤20), 24 (12.6%) were partial responders, 24 (12.6%) were relapsers and 56 (29.3%) were non‐responders. Of 87 patients categorised as responders, 81 (93.1%) sustained their clinical response and 47 (46.0%) achieved complete response. Factors associated with treatment response included female sex and less extensive and shorter duration of hair loss.

**Conclusions:**

Approximately 45% of patients were SALT score responders, with up to 11% requiring >1 year of ritlecitinib treatment to achieve response, highlighting the importance of extended treatment duration.

**ClinicalTrials.gov Registration:**

ALLEGRO phase 2b/3 study (NCT03732807); ALLEGRO‐LT study (NCT04006457).

## Graphical abstract

A post hoc analysis of Severity of Alopecia Tool (SALT) score trajectories in patients with alopecia areata who received ritlecitinib 50 mg once‐daily in ALLEGRO‐2b/3 and rolled over into the ongoing, open‐label, Phase 3 ALLEGRO‐LT study. Long‐term response patterns up to Month 24 and variables associated with treatment response are described.

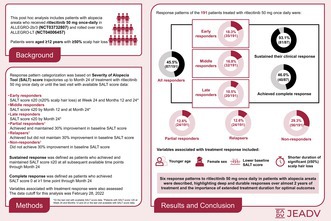

**Patterns of clinical response in patients with alopecia areata treated with ritlecitinib in the ALLEGRO clinical development programme** by King et al.

Video

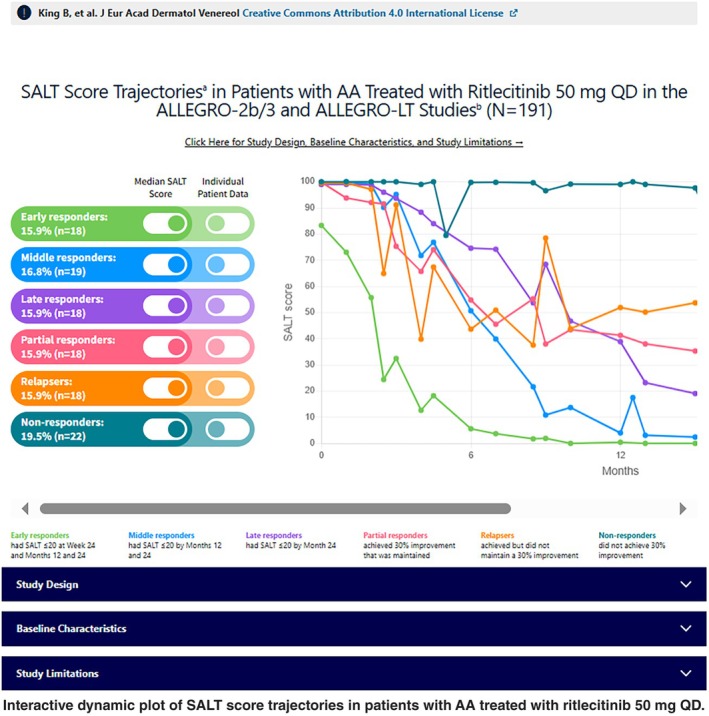

**Why was the study undertaken?**

- Alopecia areata (AA) can be chronic and progress to severe forms like alopecia totalis or alopecia universalis affecting quality of life, yet limited literature exists on the long‐term clinical response patterns to treatment, which is crucial for guiding clinical decisions.

**What does the study add?**

- Six distinct and clinically relevant response patterns were identified in patients with AA treated with ritlecitinib 50 mg daily, revealing deep and durable responses over nearly 2 years.
- Some patients achieved SALT ≤20 responses only after more than 1 year.
- Female sex, less extensive and shorter duration of hair loss were associated with a higher likelihood of a response to ritlecitinib treatment.

**What are the implications of this study for disease understanding and/or clinical care?**

- Some patients with AA may require over a year of treatment with ritlecitinib to achieve significant hair regrowth, highlighting the importance of extended treatment duration and avoiding premature therapy discontinuation for optimal outcomes.
- While initiating treatment before the onset of severe hair loss and early in an episode of severe disease may enhance the chances of hair regrowth, further research is needed to confirm this and to determine the most effective intervention timing.

## INTRODUCTION

Alopecia areata (AA) is an autoimmune disease characterised by nonscarring hair loss ranging from small alopecic patches to complete loss of scalp, face and/or body hair,^1^ and is estimated to affect approximately 2% of the global population.^2^ Between 4.5% and 36.1% of patients with AA may experience progression to extensive forms of the disease, including alopecia totalis (AT, characterised by complete scalp hair loss) or alopecia universalis (AU, involving complete loss of scalp, face and body hair).^3^, ^4^, ^5^ AA is associated with impairment of quality of life and patients may experience anxiety and/or depression.^6^, ^7^, ^8^, ^9^

The pathogenesis of AA involves the loss of immune privilege at the hair follicle and recognition of exposed hair follicle autoantigens by T‐cell receptors on autoreactive CD8^+^ T cells.^10^, ^11^, ^12^ Interferon‐γ and interleukin‐15, considered important drivers of AA, transduce signals through the Janus kinase (JAK)–signal transducer and activator of transcription (STAT) signalling pathway and are involved in the activation and proliferation of autoreactive T cells.^13^, ^14^, ^15^ Downstream signalling by exposed hair follicle autoantigens via T‐cell receptors involves the tyrosine kinase expressed in hepatocellular carcinoma (TEC) family of kinases, which have also been implicated in the pathogenesis of AA.^15^, ^16^, ^17^, ^18^

Two oral treatments for AA have recently been approved: baricitinib, a JAK1/2 inhibitor approved to treat adults with severe AA,^19^ and ritlecitinib, a selective dual JAK3/TEC family kinase inhibitor approved for the treatment of severe AA in patients aged ≥12 years.^20^ In the ALLEGRO phase 2b/3 study (ALLEGRO‐2b/3), ritlecitinib demonstrated efficacy and an acceptable safety profile at up to 48 weeks in patients aged ≥12 years with AA.^21^

Limited literature exists concerning the overall temporal pattern of clinical response to treatment in patients with AA.^22^ Such information may be important in guiding clinical decisions and effectively managing patient expectations. This analysis of the pivotal ALLEGRO‐2b/3 study and the ongoing, open‐label, Phase 3 ALLEGRO‐LT study describes trajectories of clinical response over 24 months from treatment initiation in patients with AA who received ritlecitinib 50 mg, with the goal of describing long‐term response patterns and associated baseline characteristics.

## MATERIALS AND METHODS

### Study design

**FIGURE 1:**
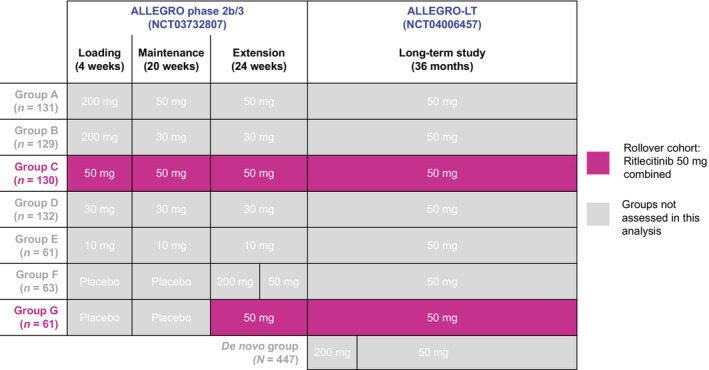
Analysis populations. Data while patients were receiving placebo were not included in this analysis; data from patients in Group G were re‐baselined from the start of treatment with ritlecitinib.

This post hoc analysis included pooled data from the pivotal, international, randomised, double‐blind, placebo‐controlled, dose‐ranging ALLEGRO‐2b/3 study (NCT03732807) and the long‐term, open‐label, Phase 3 ALLEGRO‐LT study (NCT04006457). The design and primary results of ALLEGRO‐2b/3 have been previously described.^21^ ALLEGRO‐LT is ongoing and has enrolled patients in two arms: (1) rollover patients who received study intervention in the ALLEGRO phase 2a study (NCT02974868) or ALLEGRO‐2b/3 and (2) de novo patients who had not received treatment in any other ALLEGRO study (Figure 1). The data cut‐off for this analysis was 28 February 2022. Interim results are subject to change as additional data are collected and analysed in the ongoing ALLEGRO‐LT study.

### Analysis populations

This analysis included patients from the rollover cohort who received ritlecitinib 50 mg once daily (QD) in ALLEGRO‐2b/3, including those who received placebo for the initial 24 weeks. These patients subsequently rolled over into ALLEGRO‐LT, where they continued to receive ritlecitinib 50 mg QD for up to 36 additional months (Figure 1). Patients were aged ≥12 years and had ≥50% scalp hair loss, including AT and AU (defined as baseline SALT score 100 and designated AT or AU by the investigator), with current episode of hair loss ≤10 years. Neither study had an upper age limit exclusion criterion. Exclusion criteria applied to patients who received other systemic treatments that could affect AA within 8 weeks of the first dose of the study drug or within five half‐lives of that systemic treatment, whichever was longer.^23^

### Assessment schedules and re‐baselining

To align time points within and across groups for summarisation, visit schedules were calculated as time since the first ritlecitinib dose and groups were re‐baselined. Since the visit schedule for ALLEGRO‐2b/3 was based on weeks and the schedule for ALLEGRO‐LT was based on months, 1 month in ALLEGRO‐LT was considered equivalent to 4 weeks in ALLEGRO‐2b/3, and all data are reported in months.

### Outcomes

Severity of Alopecia Tool (SALT) score trajectories were generated for individual patients up to Month 24 of treatment with ritlecitinib or until the last visit with available SALT score data. Trajectories that did not extend to Month 24 reflect patients who had not reached this time point at the data cut‐off or who had discontinued from the study. Patients were categorised into six mutually exclusive groups based on response trajectories. Patients achieving a SALT score ≤20, indicating ≤20% scalp hair loss, were considered responders. These patients were then categorised as early (SALT score ≤20 at Week 24 and Months 12 and 24), middle (SALT score ≤20 by Month 12 and at Month 24) or late responders (SALT score ≤20 by Month 24). Patients who did not achieve a SALT score of ≤20 were categorised as partial responders (achieved 30% improvement that was maintained), relapsers (achieved but did not maintain a 30% improvement) or non‐responders (did not achieve 30% improvement). Definitions for each responder group are shown in Table S1. The proportions of patients who achieved sustained response (achieved and then maintained SALT score ≤20 at all subsequent available time points through Month 24, where ‘available’ indicates that the SALT score was not missing) and complete response (SALT score 0 at ≥1 time point through Month 24) were also assessed. Patient demographics and baseline disease characteristics were assessed by response pattern.

### Statistical analysis

Analyses were conducted in the full set of patients treated with ritlecitinib 50 mg, irrespective of treatment duration, using as‐observed data. Day 1 was defined as the first day of ritlecitinib 50 mg treatment. A multivariable logistic regression using all variables, forward selection, backward elimination and stepwise selection methods was used to assess the association of patient demographics, baseline disease characteristics and the presence of comorbidities with the likelihood of achieving response (combining early, middle and late responders) or non‐response (using the definitions in Table S1); patients in the relapser and partial responder groups were excluded from this analysis. Variables used as covariates in the model included age (continuous), sex (male vs. female), race (White vs. other), body mass index (continuous), current AA episode duration (defined as the time since the patient last had substantial scalp hair, regardless of whether that hair growth occurred spontaneously or was the result of interventional treatment, with a maximum duration of ≤10 years; continuous), AA disease duration (continuous), duration of significant (≥50%) scalp hair loss (continuous), SALT score at baseline (continuous), eyelash assessment (ELA) scores at baseline, eyebrow assessment (EBA) scores at baseline, hair loss pattern (AU vs. AT, other vs. AT), number of episodes of AA, prior drug treatment for AA (yes vs. no) and comorbid conditions (including asthma, autoimmune thyroiditis, atopic dermatitis and allergic rhinitis). All analyses were implemented using R software (glm function of the stats package for logistic regressions); odds ratios and 95% confidence intervals are reported. Quartile differences (Q3–Q1) were used to quantify the odds ratio per increase in quartile difference for significant continuous covariates to better contextualise how variations in these covariates impacted the likelihood of treatment response.

## RESULTS

### Patients

A total of 191 patients were part of the rollover cohort, which comprised 130 patients who received ritlecitinib 50 mg QD throughout ALLEGRO‐2b/3 and ALLEGRO‐LT and 61 patients who received placebo for 24 weeks in ALLEGRO‐2b/3 before switching to ritlecitinib 50 mg QD. Among patients in the ritlecitinib 50‐mg combined group, mean (standard deviation) age was 33.2 (14.4) years and 107 (56.0%) were female. Abnormal EBA and ELA scores at baseline were observed in 154 (80.6%) and 139 (72.8%) patients, respectively, and 78 (40.8%) had AT or AU. The overall mean SALT score at baseline was 90.8, and the mean current AA episode duration was 3.3 years (Table 1).

**TABLE 1:** Baseline demographic and disease characteristics in rollover patients treated with ritlecitinib 50 mg QD.

	Ritlecitinib 50 mg QD combined (*N* = 191)
Age, mean (SD), years	33.2 (14.4)
12–17 years, *n* (%)	27 (14.1)
≥18 years, *n* (%)	164 (85.9)
Female, *n* (%)	107 (56.0)
Race, *n* (%)	
White	123 (64.4)
Other	68 (35.6)
BMI, mean (SD), kg/m^2^	24.9 (5.8)
Type of AA, *n* (%)	
AT^a^	37 (19.4)
AU^a^	41 (21.5)
Other	113 (59.2)
Baseline SALT score	
Mean (SD)	90.8 (14.1)
SALT score ≥ 50, *n* (%)	191 (100)
Abnormal EBA score at baseline, *n* (%)^b^	154 (80.6)
Abnormal ELA score at baseline, *n* (%)^b^	139 (72.8)
Duration of AA since diagnosis, mean (SD), years	9.8 (10.5)
Duration of current AA episode, mean (SD), years	3.3 (2.8)
Duration of significant (>50%) scalp hair loss, mean (SD), years	2.8 (2.7)
Prior pharmacological treatment for AA, *n* (%)	145 (75.9)
Comorbid conditions, *n* (%)	
Asthma	22 (11.5)
Autoimmune thyroiditis	11 (5.8)
Atopic dermatitis	31 (16.2)
Allergic rhinitis	20 (10.5)

Abbreviations: AA, alopecia areata; AT, alopecia totalis; AU, alopecia universalis; BMI, body mass index; EBA, eyebrow assessment; ELA, eyelash assessment; QD, once daily; SALT, Severity of Alopecia Tool.

^a^ Participants in the AT and AU categories had a SALT score of 100 (complete scalp hair loss) at baseline and a clinical diagnosis of AT or AU by the investigator.

^b^ Patients with abnormal EBA or ELA scores had a score of 0–2 (no eyebrows/eyelashes to moderate eyebrows/eyelashes).

### Clinical response patterns

**FIGURE 2:**
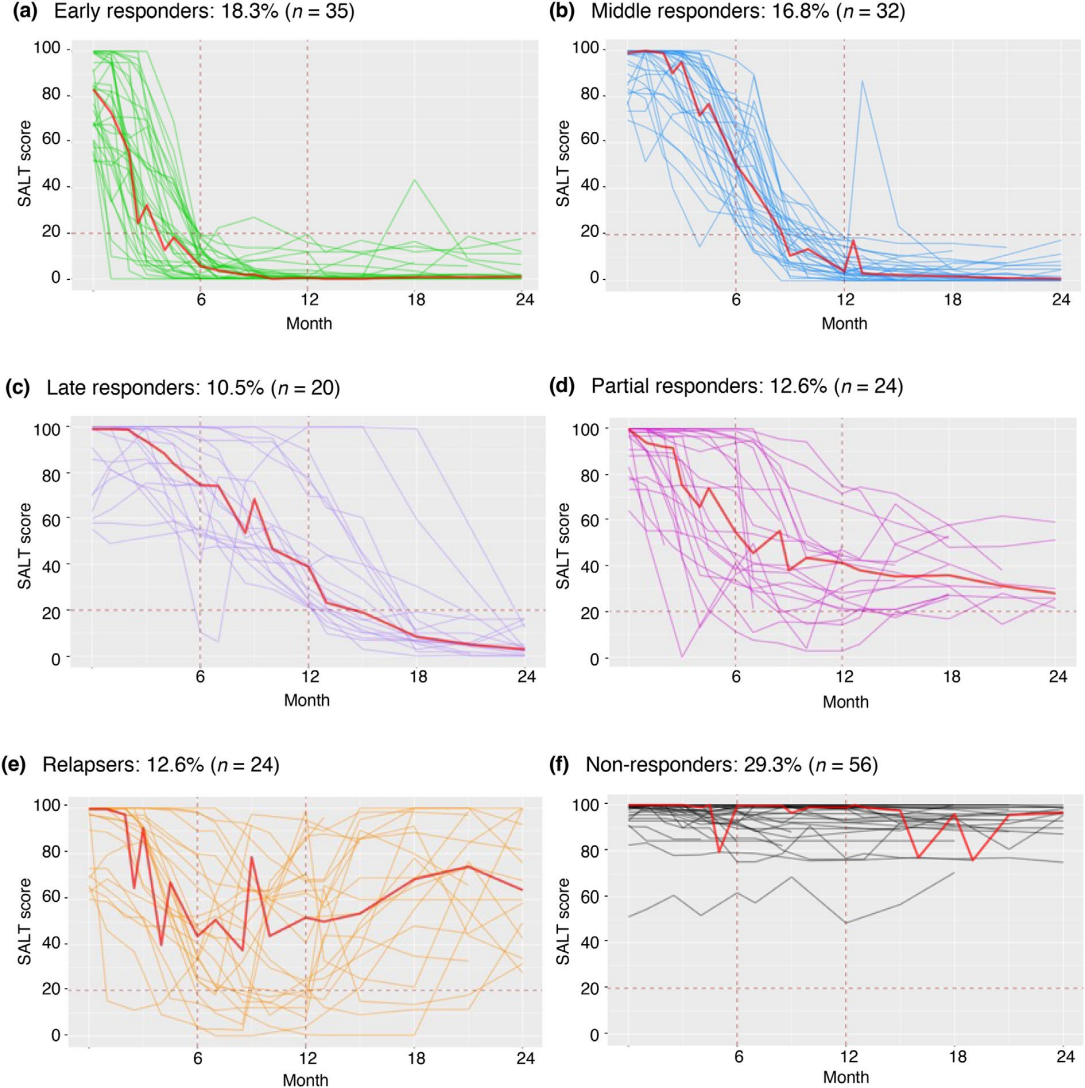
SALT score trajectories in the rollover cohort treated with ritlecitinib 50 mg QD (*N* = 191). QD, once daily; SALT, Severity of Alopecia Tool. Red lines indicate the median SALT score trajectory. Trajectories that do not extend to Month 24 reflect patients who had not reached this time point at the data cut‐off (February 2022) or who were discontinued from the study. Data are as‐observed. SALT score trajectories are shown in the Interactive dynamic plot.

**FIGURE 3:**
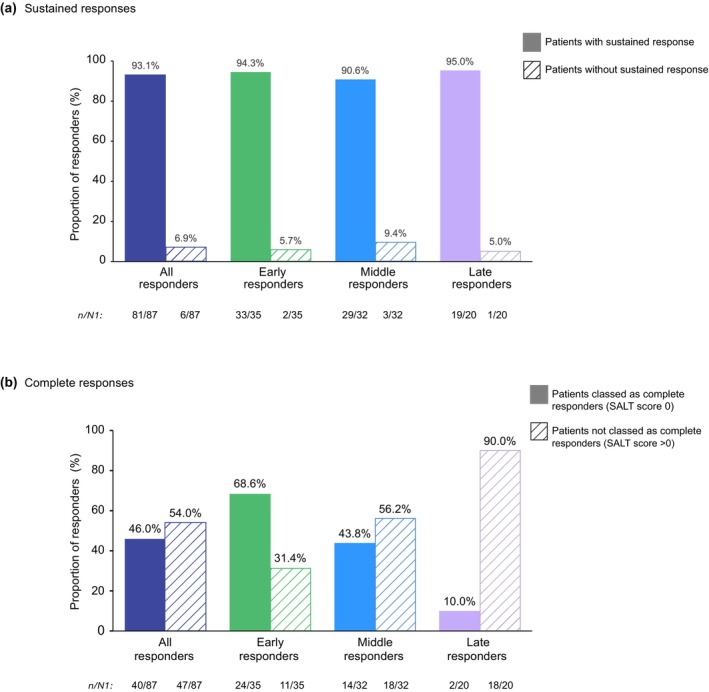
Proportions of rollover patients treated with ritlecitinib 50 mg QD who achieved SALT score ≤20 and (a) sustained response and (b) complete response. QD, once daily; SALT, Severity of Alopecia Tool. Percentages are based on n/N1, where *n* = the number of patients with sustained or complete response, and N1 = total number of patients who were classed as responders. Patients with sustained response achieved and then maintained a SALT score ≤20 at all subsequent available time points through Month 24, where ‘available’ indicates that the SALT value was not missing. Patients who never achieved SALT score ≤20 and patients who achieved SALT score ≤20 but a score of >20 at subsequent time points were excluded. Patients classed as complete responders achieved a SALT score 0 at ≥1 time point through Month 24. Data are as‐observed.

Of the 191 patients, 45.5% (87/191) were responders through Month 24; 18.3% (35/191) were early, 16.8% (32/191) were middle and 10.5% (20/191) were late responders (Figure 2a–c). There were 24 (12.6%) partial responders, 24 (12.6%) relapsers and 56 (29.3%) non‐responders (Figure 2d–f). Of the 87 responders, 46.0% (40/87) were complete responders and 93.1% (81/87) sustained their clinical response (Figure 3). Median SALT scores decreased through Month 24 in all responder groups. In early responders, the median SALT score reached 0, indicative of complete response, by Month 12. For middle responders, the median SALT score was also very low and approached 0 by Month 12. SALT score trajectories are shown in the Interactive dynamic plot.

Compared with other responder groups, early responders were predominantly female (91.4%) with less severe AA at baseline, characterised by lower mean baseline SALT score (79.9). The early responder group also included a lower proportion of patients with AT or AU (14.3%) and a lower proportion of patients with abnormal EBA or ELA scores at baseline (60.0% and 57.1%, respectively) than were present in the overall population (Table 2). Mean disease and current AA episode durations were lower in early responders (8.7 and 2.3 years, respectively) than in non‐responders (11.1 and 4.1 years, respectively).

**TABLE 2:** Baseline demographic and disease characteristics in rollover patients treated with ritlecitinib 50 mg QD (*N* = 191) by response status.

	Early responder (*n* = 35)	Middle responder (*n* = 32)	Late responder (*n* = 20)	Partial responder (*n* = 24)	Relapser (*n* = 24)	Non‐responder (*n* = 56)
Age, mean (SD), years	32.1 (13.0)	31.0 (13.9)	34.6 (14.4)	33.5 (14.7)	29.0 (13.0)	36.4 (15.7)
Female, *n* (%)	32 (91.4)	21 (65.6)	12 (60.0)	10 (41.7)	13 (54.2)	19 (33.9)
Race, *n* (%)						
White	21 (60.0)	18 (56.2)	18 (90.0)	13 (54.2)	12 (50.0)	41 (73.2)
Other	14 (40.0)	14 (43.8)	2 (10.0)	11 (45.8)	12 (50.0)	15 (26.8)
BMI, mean (SD), kg/m^2^	24.4 (6.3)	24.0 (4.8)	23.9 (5.1)	25.5 (9.1)	24.3 (5.1)	26.1 (4.5)
Type of AA, *n* (%)						
AT^a^	2 (5.7)	5 (15.6)	3 (15.0)	6 (25.0)	2 (8.3)	19 (33.9)
AU^a^	3 (8.6)	9 (28.1)	6 (30.0)	5 (20.8)	5 (20.8)	13 (23.2)
Other	30 (85.7)	18 (56.2)	11 (55.0)	13 (54.2)	17 (70.8)	24 (42.9)
Baseline SALT score, mean (SD)	79.9 (17.0)	93.4 (9.5)	87.5 (17.3)	93.6 (10.4)	89.5 (15.3)	96.8 (9.4)
Abnormal EBA score at baseline, *n* (%)^b^	21 (60.0)	26 (81.3)	17 (85.0)	22 (91.7)	19 (79.2)	49 (87.5)
Abnormal ELA score at baseline, *n* (%)^b^	20 (57.1)	21 (65.6)	16 (80.0)	19 (79.2)	16 (66.7)	47 (83.9)
Duration of AA since diagnosis, mean (SD), years	8.7 (11.4)	8.6 (9.2)	10.8 (12.8)	10.7 (10.4)	8.5 (6.6)	11.1 (11.3)
Duration of current AA episode, mean (SD), years	2.3 (2.2)	2.5 (2.4)	3.1 (2.7)	4.0 (3.0)	3.1 (2.3)	4.1 (3.1)
Duration of significant (≥50%) scalp hair loss, mean (SD), years	2.1 (2.6)	2.2 (2.1)	1.9 (2.0)	3.2 (2.7)	3.1 (2.6)	3.6 (3.0)
Prior pharmacological treatment for AA, *n* (%)	29 (82.9)	25 (78.1)	15 (75.0)	16 (66.7)	20 (83.3)	40 (71.4)
Comorbid conditions, *n* (%)						
Asthma	4 (11.4)	1 (3.1)	1 (5.0)	3 (12.5)	5 (20.8)	8 (14.3)
Autoimmune thyroiditis	2 (5.7)	2 (6.2)	1 (5.0)	4 (16.7)	0	2 (3.6)
Atopic dermatitis	7 (20.0)	5 (15.6)	1 (5.0)	5 (20.8)	7 (29.2)	6 (10.7)
Allergic rhinitis	5 (14.3)	4 (12.5)	1 (5.0)	5 (20.8)	3 (12.5)	2 (3.6)

*Note*: Grey cells indicate responder populations. Abbreviations: AA, alopecia areata; AT, alopecia totalis; AU, alopecia universalis; BMI, body mass index; EBA, eyebrow assessment; ELA, eyelash assessment; QD, once daily; SALT, Severity of Alopecia Tool.

^a^ Participants in the AT and AU categories had a SALT score of 100 (complete scalp hair loss) at baseline and a clinical diagnosis of AT or AU by the investigator.

^b^ Patients with abnormal EBA or ELA scores had a score of 0–2 (no eyebrows/eyelashes to moderate eyebrows/eyelashes).

**FIGURE 4:**
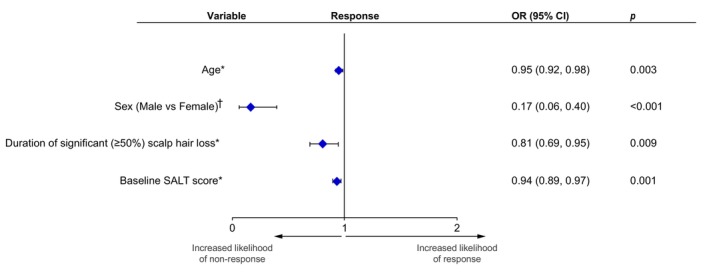
Association of patient demographics, baseline disease characteristics and comorbidities with treatment response in rollover patients treated with ritlecitinib 50 mg QD. CI, confidence interval; OR, odds ratio; QD, once daily; SALT, Severity of Alopecia Tool. Multivariable logistic regression models evaluated the non‐responder and responder (SALT score ≤20, irrespective of early, middle and late response) populations only; patients in the relapser and partial responder groups were excluded from this analysis. Independent covariates of interest that were significant in the stepwise models are shown. *Continuous variable in which a change in the variable (1 year for age, duration of significant [≥50%] scalp hair loss, episode duration and disease duration; 1 unit for baseline SALT score) is associated with the likelihood of response. ^†^The latter is the reference category.

The baseline disease characteristics of male and female patients were investigated. A higher proportion of male patients had AT or AU, more extensive hair loss and longer durations of both the current AA episode and significant (≥50%) scalp hair loss at baseline (Table S2). Of the independent covariates analysed in multivariable logistic regression models, younger age, female sex, shorter duration of significant (≥50%) scalp hair loss and lower baseline SALT score were independent factors associated with increased likelihood of treatment response versus non‐response (Figure 4; Table S3). Holding all other variables constant, a 21‐year increase in age would reduce the likelihood of response by 63.6%. A 3‐year increase in duration of significant (≥50%) scalp hair loss would reduce the likelihood of response by 46.5%, while a 15‐unit increase in baseline SALT score would reduce the likelihood of response by 63.5% (Table S4). However, patients achieving a SALT score ≤20 were observed across all three response categories (Figure 2a–c). The presence of atopic comorbidities and autoimmune thyroiditis was not associated with treatment response (Table S3).

## DISCUSSION

In this post hoc analysis in rollover patients in the ALLEGRO‐LT study who received the approved 50‐mg dose of ritlecitinib, trajectories of clinical response include six distinct and clinically relevant response patterns. This population exhibited severe disease at baseline, characterised by a high mean SALT score and had an average current AA episode duration of approximately 3 years; over 70% of patients had eyebrow and/or eyelash hair loss. Of the 191 patients, 45.5% achieved a response through 2 years of treatment. The low median SALT scores observed, particularly in early and middle responders, indicate that several patients achieved full or almost full hair regrowth. Notably, 10.5% of patients achieved response only after >1 year of treatment, suggesting the potential benefit of extended treatment for those with an initial suboptimal response and emphasizing the importance of avoiding premature treatment discontinuation. Of patients who achieved a SALT score ≤20 response in this study, over 90% sustained a SALT score ≤20 and 46% achieved a complete response (SALT score 0), indicating full hair regrowth at ≥1 time point through 24 months. These data highlight a substantial and consistent treatment effect of ritlecitinib 50 mg over an extended period (>1 year).

Interim results from the ALLEGRO‐LT study, based on the most recent data cut‐off, support the potential of extended treatment with ritlecitinib, as the proportion of patients who achieved SALT score ≤20 responses continued to increase from Month 12 to Month 24, including among patients with AT/AU.^24^ While extended treatment with ritlecitinib may have potential benefits in difficult‐to‐treat patients, including refractory AT/AU, it is important for physicians to manage treatment expectations as outcomes may vary. Given the higher discontinuation rate observed among patients with AT/AU, primarily due to a lack of efficacy, compared with patients without AT/AU,^24^ it is important to inform patients about their likelihood of achieving a sustained SALT ≤20 response over 24 months.

Approximately 13% of patients were categorised as partial responders, and 13% of patients were relapsers; some of the partial responders and relapsers achieved a SALT score ≤20 that was not subsequently maintained throughout the study period. In clinical practice, where adjunctive therapies may be considered in combination with JAK inhibitors, there is potential for partial responders to achieve and sustain SALT score ≤20. In a small case series, the use of combined systemic corticosteroids and baricitinib led to rapid hair regrowth in all patients with AA, suggesting that such combination therapy could accelerate positive outcomes and potentially reduce the time required to observe SALT score ≤20.^25^ Further research, including randomised controlled trials, is needed to evaluate the benefit–risk ratio of combination therapies for AA.

Early responders comprised patients with less severe AA at baseline. These patients had lower mean baseline SALT scores, and there were lower proportions of patients with AT or AU and abnormal EBA or ELA scores than the overall study population. Analysis of response trajectories in patients treated with baricitinib for up to 52 weeks in the BRAVE‐AA studies also showed that early response (defined as ≥30% improvement from baseline in SALT score by Week 12) was more frequent among patients with lower baseline SALT scores (50–94) compared with those with very severe hair loss (SALT score 95–100).^22^ Less extensive hair loss (lower baseline SALT scores) was significantly associated with increased likelihood of treatment response in this analysis of ritlecitinib. These data add to a growing body of evidence indicating that patients with more severe hair loss (higher SALT scores), in particular those with AT or AU, respond less well to treatment compared with patients with more limited disease.^26^, ^27^, ^28^, ^29^, ^30^, ^31^, ^32^, ^33^, ^34^ Additionally, in the present analyses, shorter duration of significant (≥50%) scalp hair loss was significantly associated with treatment response. This is consistent with previous studies indicating that patients with long‐standing AA tend to be more refractory to treatment.^27^, ^29^, ^30^ Longer duration of current AA episode (≥4 years) and more extensive AA (SALT score 95–100) were more common among non‐responders in a 52‐week analysis of baricitinib 2 and 4 mg.^22^ In a previous study of the JAK inhibitor tofacitinib in AA, treatment response was negatively correlated with the duration of the current episode of AA.^30^ AA episode duration of <1 year was found to be significantly associated with SALT score ≤20 response in a post hoc analysis of the ALLEGRO‐2b/3 study.^35^ Together, these analyses suggest that intervention before very severe hair loss occurs and early in an episode of severe disease may improve the chances of treatment success, particularly in cases where the disease shows resistance or significant progression. This is consistent with European expert consensus on the systemic treatment of AA, which underscores that a ‘wait‐and‐see’ approach is not advisable for patients eligible for systemic therapy given the low chance of spontaneous hair regrowth and the better response to systemic therapy in patients with a shorter disease duration.^36^

A new finding in this study is a disproportionately greater treatment response in female patients compared with male patients. Although more women than men were enrolled in the ALLEGRO‐2b/3 study (446 vs. 272, respectively),^21^ men had more severe disease at baseline, which may account for the lower treatment efficacy in these patients. Concomitant unrecognised androgenetic alopecia in men, which could prevent achievement of SALT score ≤20, may also explain the disproportionate response in women.

Previous studies have reported an association between AA and atopic diseases, particularly atopic dermatitis, allergic rhinitis and asthma.^37^, ^38^, ^39^ Indeed, in this analysis, of the 191 patients, 38.2% had at least one atopic disease and 5.8% had autoimmune thyroiditis. The presence or absence of atopic comorbidities or autoimmune thyroiditis was not associated with clinical response to ritlecitinib treatment.

This analysis is not without limitations. Response pattern definitions in this analysis diverge from the clinical endpoint assessments conducted in ALLEGRO‐2b/3,^21^ and because the analyses in this study are of as‐observed data, the proportions of responders cannot be directly compared with those published previously. Acknowledging that treatment success in AA is ultimately patient‐specific and may depend on factors such as sex, hair style and cultural background, there is no clinical consensus on this matter. While SALT score ≤20 may be considered a therapeutic goal,^40^ further improvement to SALT score ≤10 may be necessary to enhance quality of life.^41^ Additionally, the small patient numbers in certain response groups may limit the interpretation of these results. The ALLEGRO‐LT study is ongoing and will provide additional data on long‐term response patterns in patients with AA treated with ritlecitinib. The full safety profile of ritlecitinib across the Phase 2 and 3 studies of the ALLEGRO clinical programme has been reported previously and supports the chronic use of ritlecitinib in patients aged ≥12 years with AA.^42^

In conclusion, these analyses described six response patterns among patients with AA treated with ritlecitinib 50 mg QD and revealed deep and durable responses over almost 2 years of treatment, although some patients achieved SALT score ≤20 responses only after >1 year of treatment. Of the 191 patients, 45.5% achieved a response and 93.1% of these responders sustained this response through 24 months. Patient demographics and baseline disease characteristics, including female sex, less extensive scalp hair loss and shorter duration of hair loss, were found to be associated with an increased likelihood of response to ritlecitinib treatment. These analyses highlight the importance of extended treatment duration and avoiding premature therapy discontinuation for optimal outcomes. While initiating ritlecitinib treatment before the onset of very severe hair loss and early in an episode of severe disease may enhance the chances of hair regrowth, further research is needed to confirm this possibility and to determine the most effective timing for intervention.

## AUTHOR CONTRIBUTIONS

UK, DW, RIA, SHZ and AL contributed to the concept and design of the study. All authors contributed to data analysis and interpretation and critical revision of the publication, and all authors read and approved the final manuscript.

## FUNDING INFORMATION

This study was sponsored by Pfizer, Inc.

## CONFLICT OF INTEREST STATEMENT

BK has served on advisory boards, is a consultant, is a clinical trial investigator and/or is on a data monitoring committee for AbbVie, AltruBio, Inc., Almirall, AnaptysBio, Arena Pharmaceuticals, Aslan Pharmaceuticals, Bioniz Therapeutics, Bristol Meyers Squibb, Concert Pharmaceuticals, Inc., Equillium, Horizon Therapeutics, Eli Lilly and Company, Incyte Corp, Janssen Pharmaceuticals, LEO Pharma, Merck, Otsuka/Visterra, Inc., Pfizer, Inc., Q32 Bio, Inc., Regeneron, Sanofi Genzyme, Sun Pharmaceutical, TWi Biotechnology, Inc., Viela Bio and Ventyx Biosciences, Inc.; and has served on speakers bureaus for AbbVie, Incyte, Eli Lilly, Pfizer, Regeneron and Sanofi Genzyme. PM received investigator grants and/or research funding from Concert Pharmaceuticals, Pfizer, Inc. and Eli Lilly. KL served as an investigator, advisory board member and consultant for Pfizer, received educational grant funding from Pfizer and has been a consultant for Aquis. YR served as consultant, speaker, or investigator for AbbVie, Bristol Myers Squibb, Boehringer Ingelheim, Pfizer, Pepticom, Novartis, Janssen, Neopharm, Giuliani S.p.A, Dexcel Pharma, Eli Lilly, Sanofi, Taro and Monasterium Laboratory; and serves as the CMO of MII Labs. RS has provided professional services to Aerotech, AbbVie, AstraZeneca, Akesobio, Amgen, Arcutis, Arena, Ascend, Bayer, BMS, Boehringer Ingelheim, Celgene, Coherus BioSciences, Connect, Cutanea, Demira, Eli Lilly, Galderma, GSK, Janssen, LEO Pharma, MedImmune, Merck, MSD, Novartis, Oncobiologics, Pfizer, Regeneron, Reistone, Roche, Samson Clinical, Sanofi, Sun Pharma and UCB. LA has served as a consultant for AbbVie and L'Oreal. KE has served as a consultant for and/or received investigator grants from Abbvie, Almirall, Incyte, L'Oreal, La Roche Posay, Pfizer, Pierre Fabre, Sanofi and Viela Bio. CP has served as a consultant for Almirall, Amgen, AbbVie, Apogee Therapeutics, BMS, Boehringer Ingelheim, Celgene, Galderma, GSK, Eli Lilly, IQVIA, Janssen, Leo Pharma, Merck, Mylan, Novartis, Pfizer, Pierre Fabre, Sanofi and UCB Pharma. MO is a medical advisor for and receives advisory fees from Pfizer Japan, Inc., Taisho Pharmaceutical Co, Eli Lilly Japan KK, ROHTO Pharmaceutical Co, Bristol Myers Squibb Japan and AbbVie GK; receives lecture fees from Eli Lilly Japan KK and Pfizer Japan, Inc.; and receives research grants for projects not related to this study from Maruho Co, Sun Pharma Japan Ltd., Advantest Corp and Shiseido Co. RAE is an employee of Health Services Consulting Corporation and received consultancy fees from Pfizer in connection with this study. GB is an employee of Engineering Ingegneria Informatica, a paid sub‐contractor to Health Services Consulting Corporation, in conjunction with this analysis. UK, DW, RIA, SHZ and AL are employees of and hold stock or stock options in Pfizer, Inc.

## ETHICAL APPROVAL

The protocols were reviewed and approved by the institutional review boards or ethics committees of the participating institutions. The studies were conducted in accordance with the International Ethical Guidelines for Biomedical Research Involving Human Subjects (Council for International Organizations of Medical Sciences 2002), ICH Guideline for Good Clinical Practice and the Declaration of Helsinki.

## ETHICS STATEMENT

Written informed consent was obtained from each patient, parent or the patient's legal representative.

## Supporting information


Table S1.

Table S2.

Table S3.

Table S4.

## Data Availability

Upon request, and subject to review, Pfizer will provide the data that support the findings of this study. Subject to certain criteria, conditions and exceptions, Pfizer may also provide access to the related individual de‐identified participant data. See https://www.pfizer.com/science/clinical‐trials/trial‐data‐and‐results for more information.
