# Supplementary material for: Patterns of clinical response in patients with alopecia areata treated with ritlecitinib in the ALLEGRO clinical development programme
Source: J Eur Acad Dermatol Venereol. 2025 Feb 17;39(6):1163–73. doi: 10.1111/jdv.20547 (PMC12105426; doi:10.1111/jdv.20547)
Supplement: Supplementary file 1 — Table S1. [file JDV-39-1163-s001.docx]

**Table S1.** Classification and definitions of SALT score trajectories and other definitions used in this analysis

| **Category** | **Definition** |
| --- | --- |
| **Response patterns** | |
| **Early responder** | Patients who achieved SALT score ≤20 (≤20% scalp hair loss) at Week 24 and Months 12 and 24*^†^ |
| **Middle responder** | Patients who did not achieve SALT score ≤20 by Week 24 but did so by Month 12 and at Month 24*^†^ |
| **Late responder** | Patients who did not achieve SALT score ≤20 by Month 12 but did so by Month 24*^†^ |
| **Partial responder** | Patients with SALT score >20 at Week 24 and Months 12 and 24*^†^ who achieved 30% improvement in SALT score from baseline that was maintained thereafter |
| **Relapser** | Patients with SALT score >20 at Week 24 and Months 12 and 24*^†^ who achieved 30% improvement in SALT score from baseline that was not maintained |
| **Non-responder** | Patients with SALT score >20 at Week 24 and Months 12 and 24*^†^ who did not achieve 30% improvement in SALT score from baseline |
| **Other definitions** | |
| **Sustained response** | Patients who achieved and then maintained SALT score ≤20 at all subsequent available time points through Month 24, where “available” indicates that the SALT value is not missing (excludes patients who never achieved SALT score ≤20 and patients who achieved SALT score ≤20 but had SALT score >20 at subsequent time points) |
| **Complete response** | Patients who achieved SALT score 0 at ≥1 time point through Month 24 |

SALT, Severity of Alopecia Tool.

*Or the last visit with available SALT score data.

^†^The ALLEGRO phase 2b/3 study used weeks as the time frame for data analysis, while the ALLEGRO-LT study used months. Each month in the ALLEGRO-LT study was converted into 4 weeks to align the time frames across the 2 studies.
