# Supplementary material for: Patterns of clinical response in patients with alopecia areata treated with ritlecitinib in the ALLEGRO clinical development programme
Source: J Eur Acad Dermatol Venereol. 2025 Feb 17;39(6):1163–73. doi: 10.1111/jdv.20547 (PMC12105426; doi:10.1111/jdv.20547)
Supplement: Supplementary file 2 — Table S2. [file JDV-39-1163-s003.docx]

**Table S2.** Baseline characteristics of the rollover cohort by sex

|  | **Female**  **(n=107)** | **Male**  **(n=84)** |
| --- | --- | --- |
| **Age, mean (SD), years** | 36.2 (15.1) | 29.4 (12.5) |
| 12-17 years, n (%) | 12 (11.2) | 15 (17.9) |
| ≥18 years, n (%) | 95 (88.8) | 69 (82.1) |
| **Race, n (%)** |  |  |
| White | 70 (65.4) | 53 (63.1) |
| Other | 37 (34.6) | 31 (36.9) |
| **BMI, mean (SD), kg/m^2^** | 24.4 (5.2) | 25.5 (6.4) |
| **Type of AA, n (%)** |  |  |
| AT* | 13 (12.1) | 24 (28.6) |
| AU* | 20 (18.7) | 21 (25.0) |
| Other | 74 (69.2) | 39 (46.4) |
| **Baseline SALT score, mean (SD)** | 87.8 (15.5) | 94.8 (11.0) |
| **Abnormal EBA score at baseline, n (%)^†^** | 80 (74.8) | 74 (88.1) |
| **Abnormal ELA score at baseline, n (%)^†^** | 69 (64.5) | 70 (83.3) |
| **Duration of AA since diagnosis, mean (SD), years** | 11.4 (12.3) | 7.8 (7.2) |
| **Duration of current AA episode, mean (SD), years** | 2.9 (2.6) | 3.8 (2.9) |
| **Duration of significant (≥50%) scalp hair loss, mean (SD), years** | 2.5 (2.6) | 3.1 (2.7) |
| **Prior pharmacological treatment for AA, n (%)** | 83 (77.6) | 62 (73.8) |
| **Comorbid conditions** |  |  |
| Asthma | 13 (12.1) | 9 (10.7) |
| Autoimmune thyroiditis | 10 (9.3) | 1 (1.2) |
| Atopic dermatitis | 20 (18.7) | 11 (13.1) |
| Allergic rhinitis | 14 (13.1) | 6 (7.1) |

AA, alopecia areata; AT, alopecia totalis; AU, alopecia universalis; BMI, body mass index; EBA, eyebrow assessment; ELA, eyelash assessment; SALT, Severity of Alopecia Tool.

*Participants in the AT and AU categories had a SALT score of 100 (complete scalp hair loss) at baseline and a clinical diagnosis of AT or AU by the investigator.

^†^Patients with abnormal EBA or ELA scores had a score of 0 to 2 (no eyebrows/eyelashes to moderate eyebrows/eyelashes).
