# Supplementary material for: Patterns of clinical response in patients with alopecia areata treated with ritlecitinib in the ALLEGRO clinical development programme
Source: J Eur Acad Dermatol Venereol. 2025 Feb 17;39(6):1163–73. doi: 10.1111/jdv.20547 (PMC12105426; doi:10.1111/jdv.20547)
Supplement: Supplementary file 3 — Table S3. [file JDV-39-1163-s004.docx]

**Table S3.** ORs for treatment response in rollover patients treated with ritlecitinib 50 mg QD

| **Variable** | | **Model with**  **all variables,**  **OR (95% CI); *p* value** | **Forward,**  **OR (95% CI); *p* value** | **Backward,**  **OR (95% CI); *p* value** | **Stepwise,**  **OR (95% CI); *p* value** |
| --- | --- | --- | --- | --- | --- |
| **Age in years*** |  | 0.949 (0.912, 0.984); 0.007 | 0.953 (0.923, 0.982); 0.003 | 0.953 (0.923, 0.982); 0.003 | 0.953 (0.923, 0.982); 0.003 |
| **Sex** | **Male vs**  **female** | 0.157 (0.054, 0.418); <0.001 | 0.165 (0.064, 0.397); <0.001 | 0.165 (0.064, 0.397); <0.001 | 0.165 (0.064, 0.397); <0.001 |
| **Race** | **White vs**  **other** | 1.126 (0.402, 3.176); 0.820 | . | . | . |
| **BMI in kg/m^2^*** |  | 1.015 (0.921, 1.123); 0.769 | . | . | . |
| **Episode duration**  **in years*** |  | 0.978 (0.771, 1.255); 0.854 | . | . | . |
| **Disease duration**  **in years*** |  | 0.967 (0.907, 1.028); 0.283 | . | . | . |
| **Prior**  **pharmacological**  **treatment for AA** | **Yes vs no** | 0.877 (0.251, 2.960); 0.834 | . | . | . |
| **Hair loss pattern (AT is reference category)** | **AU vs AT** | 3.296 (0.824, 14.239); 0.098 | . | . | . |
|  | **Other**  **vs AT** | 1.315 (0.331, 5.295); 0.696 | . | . | . |
| **Number of**  **episodes of AA** |  | 1.069 (0.871, 1.329); 0.533 | . | . | . |
| **Duration of significant (≥50%) scalp**  **hair loss in years*** |  | 0.859 (0.659, 1.097); 0.224 | 0.812 (0.688, 0.945); 0.009 | 0.812 (0.688, 0.945); 0.009 | 0.812 (0.688, 0.945); 0.009 |
| **SALT score at baseline*** |  | 0.915 (0.859, 0.964); 0.002 | 0.935 (0.893, 0.970); 0.001 | 0.935 (0.893, 0.970); 0.001 | 0.935 (0.893, 0.970); 0.001 |
| **ELA score at baseline** |  | 1.509 (0.713, 3.345); 0.291 | . | . | . |
| **EBA score at baseline** |  | 0.658 (0.258, 1.585); 0.361 | . | . | . |
| **Asthma** | **Yes vs no** | 0.474 (0.088, 2.263); 0.359 | . | . | . |
| **Autoimmune**  **thyroiditis** | **Yes vs no** | 1.410 (0.160, 20.398); 0.774 | . | . | . |
| **Atopic dermatitis** | **Yes vs no** | 1.998 (0.478, 9.362); 0.355 | . | . | . |
| **Allergic rhinitis** | **Yes vs no** | 3.903 (0.628, 38.225); 0.179 | 3.820 (0.702, 31.317); 0.151 | 3.820 (0.702, 31.317); 0.151 | 3.820 (0.702, 31.317); 0.151 |

AA, alopecia areata; AT, alopecia totalis; AU, alopecia universalis; BMI, body mass index; EBA, eyebrow assessment; ELA, eyelash assessment; OR, odds ratio; QD, once daily; SALT, Severity of Alopecia Tool.

Gray cells indicate the independent variables that were significantly associated with treatment response across all models.

*Continuous variable in which a 1-unit change in the variable is associated with the likelihood of response.
