# Supplementary material for: Patterns of clinical response in patients with alopecia areata treated with ritlecitinib in the ALLEGRO clinical development programme
Source: J Eur Acad Dermatol Venereol. 2025 Feb 17;39(6):1163–73. doi: 10.1111/jdv.20547 (PMC12105426; doi:10.1111/jdv.20547)
Supplement: Supplementary file 4 — Table S4. [file JDV-39-1163-s002.docx]

**Table S4.** Impact of quartile difference (Q3 – Q1) on ORs of significant continuous covariates for treatment response in rollover patients treated with ritlecitinib 50 mg QD

| **Variable** | **First quartile** | **Third quartile** | **OR (for 1-unit increase)** | **Increase from Q1 to Q3** | **OR  for an increase in Q3 – Q1** |
| --- | --- | --- | --- | --- | --- |
| **Age in years** | 22.0 | 43.0 | 0.953 | 21.0 | 0.364 |
| **Duration of significant (≥50%) scalp hair loss in years** | 0.8 | 4.0 | 0.812 | 3.2 | 0.535 |
| **SALT score at baseline** | 85.0 | 100.0 | 0.935 | 15.0 | 0.365 |

OR, odds ratio; QD, once daily; SALT, Severity of Alopecia Tool.

Quartile differences (Q3 – Q1) were used to quantify the OR per increase in quartile difference for significant continuous covariates.
